# Ventanillas de Salud (VDS) and Mobile Health Units (MHU): A binational collaborative models

**DOI:** 10.3389/fpubh.2022.976941

**Published:** 2022-11-09

**Authors:** María Gudelia Rangel Gómez, Saúl Salazar, Ana María López Jaramillo, Isaura Angélica Lira Chávez, Alejandra Romero Rangel, Martha Leticia Caballero Abraham, Luis Gutiérrez Reyes, Cecilia B. Rosales

**Affiliations:** ^1^El Colegio de la Frontera Norte, Tijuana, Mexico; ^2^US-Mexico Border Health Commission, Tijuana, Mexico; ^3^Secretariat of Health in Mexico, Mexico City, Mexico; ^4^Instituto de los Mexicanos en el Exterior, Mexico City, Mexico; ^5^Division of Public Health Practice and Translational Research, Mel and Enid Zuckerman College of Public Health, University of Arizona, Phoenix, AZ, United States

**Keywords:** migration, health, Ventanillas de Salud, Mobile Health Units, binational collaboration

## Abstract

Over the years, the Mexican population in the United States has faced high prevalence of health-related inequalities and disadvantages and represents one of the most vulnerable migrant groups in the country. To help reduce the gaps in health care for the Mexican population, the Mexican government, in collaboration with strategic allies from various sectors, launched the Ventanillas de Salud (VDS) strategy, which was subsequently reinforced through the Mobile Health Units (MHU) care model. Both the VDS strategy and the MHU care model are intended to contribute to the development of initiatives, projects, and actions in health that will benefit the Mexican community living in the United States, which lacks or has difficulty accessing health services. This article provides a descriptive, analytical analysis of the VDS strategy and the MHU care model, as unique collaborative models, which can be replicated, and have achieved a positive impact on the health of Mexican and other Hispanic communities in the United States, at both the individual and community level.

## Introduction

According to data from the Current Population Survey 2020, the Hispanic population in the United States (62.1 million people) represents 18.6% of the total population (331 million), while the community of Mexican origin (38.5 million) accounts for 11.5%. Within this Hispanic community, the majority have American citizenship (80.2% Hispanics and 81.2% Mexicans), either by birth or naturalization; one in 10 obtained Legal Permanent Resident status and one in 10 lacks documents for their legal stay in the United States. In absolute numbers, 10.8 million of all Hispanics lack medical coverage, of which 7.2 million are of Mexican origin (1). The lack of access to medical services, coupled with poor financial conditions and limited English proficiency, which are common barriers to accessing medical care for the immigrant population, have a negative impact on the quality of life and poor health status of Mexicans in the United States.

Given this outlook, and in response to needs, in 2001, a collaborative project was implemented between Mexico and the United States called Binational Health Week (BHW). This annual event takes place in October when health is promoted and access to various medical services and prevention activities is facilitated (2).

Based on the experience of the BHW, and as a result of pressure from community leaders and local organizations requesting the continuity of the services provided, in 2003, the Ventanillas de Salud (VDS) strategy was implemented as a pilot project in the Mexican Consulates in San Diego and Los Angeles, California, with the support of the United States-Mexico Border Health Commission (USMCB), the United States-Mexico Health Initiative (currently the Health Initiative of the Americas), the University of California and The California Endowment [(3), p. 19]. In 2004, the VDS strategy was formalized and extended to other consulates. Subsequently, in 2016, as part of a strategy to strengthen the VDS, the Mexican Section of the United States-Mexico Border Health Commission implemented the Mobile Health Units (MHU) care model (4).

The VDS strategy and the MHU care model are designed to facilitate access to health services and contribute to fostering a culture of self-care among the Mexican population living in the United States, and to promote disease prevention and control [(4), p. 7–14]. However, even though the programmatic goal of VDS is to serve the Mexican population living in the United States, VDS also served other Hispanic populations living in the same communities. Therefore, we use the term “Hispanic” throughout this article to refer to the population served.

## Methodology

Secondary data sources with lack of personally identifiable information were reviewed. First, the database of the Current Population Survey 2020 (1) of the United States Census Bureau, which contains a representative sample of the Hispanic community in the United States, was analyzed to determine the typical characteristics of the population (5).

The database of the System of Continuous Information and Health Reports of Mexicans in the United States (SICRESAL-MX) was consulted. This technological tool was developed by the Mexican Section of the USMBHC, which has a record of the people served, and preventive services provided by both the VDS and the MHU. This document includes the analysis of 6.4 million records of people who received twenty million services during the period from 2019–2021. The analysis was descriptive, based on simple frequencies, and cross-referenced basic sociodemographic variables.

No personally identifiable information was included in the analysis thereby avoiding the need for the participation of human subjects and the approval of ethics committees.

## Results

### Contents of the VDS strategy and the MHU care model

Ventanillas de Salud (VDS) is a Mexican Government Strategy, undertaken by the Ministry of Health (SS) and the Ministry of Foreign Affairs (SRE), through the Institute of Mexicans Abroad (IME). It is implemented in the Mexican Consular Network in the United States and operated by local agencies, with the support of strategic allies in that country, such as government organizations, civil society and private organizations and academic institutions (6).

**Map 1:**
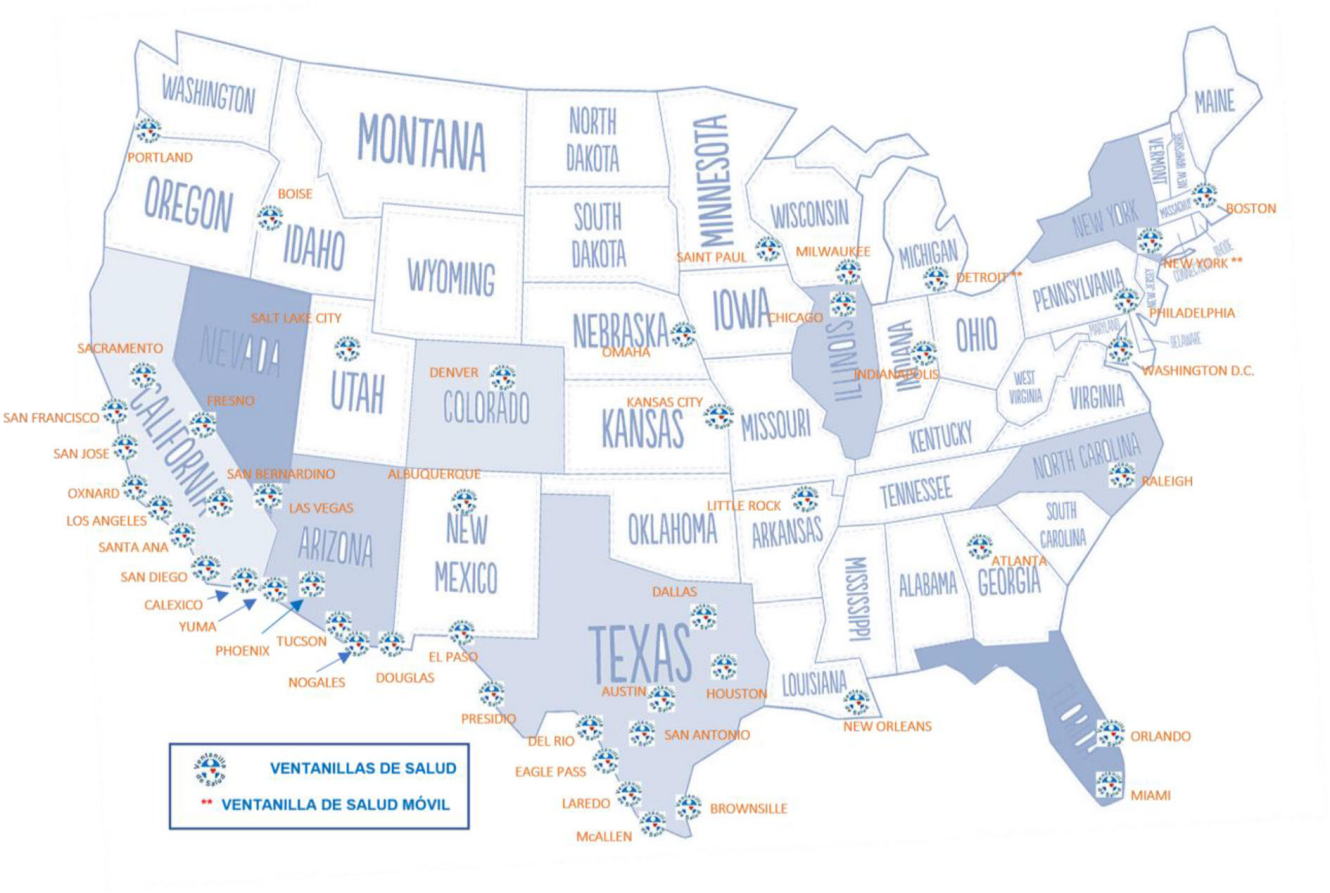
Geographical location of Ventanillas de Salud in the United States. Source: Mexican section of the Mexico-United States Border Health Commission, 2020.

The mission of VDS is to improve access to basic and preventive health services, increase public health insurance coverage, and establish a medical home, through counseling, education, timely detection, and referrals to quality health facilities, in a safe environment. Fifty-one VDS currently operate in the Mexican consular network in the US, as shown in Map 1.

VDS serves Hispanics in a situation of vulnerability who live in the United States, by promoting a sense of responsibility to improve their own health and quality of life and the acquisition of accurate health-related information. VDS facilitates access to preventive health services and fosters a culture of self-care, including active participation in health matters.

VDS has the following characteristics:

1) Services are based on the conditions that most affect the Mexican population;
2) By focusing on the individual needs of each user, they create a relationship of trust and empathy with the population served;
3) They have specialized, culturally appropriate materials to provide information in the source language;
4) They are operated by personnel trained in disease prevention and control and provide resources and options for access to health services in collaboration with community health centers and institutions, and
5) They work to improve the physical and mental health conditions of the Mexican population in the United States, and to maintain a healthy environment based on local and binational collaboration [(4), p. 8].

To fulfill its mission, VDS has over six hundred allies which include health institutions such as hospitals and federal health centers, community clinics, government organizations and educational institutions, which contribute to providing screening services, delivery of printed educational material, and help with navigating the health system in the United States, among others. These collaborative partners comprise a broad and valuable network that provides comprehensive preventive health services including health education, screening, and referrals.

**Map 2:**
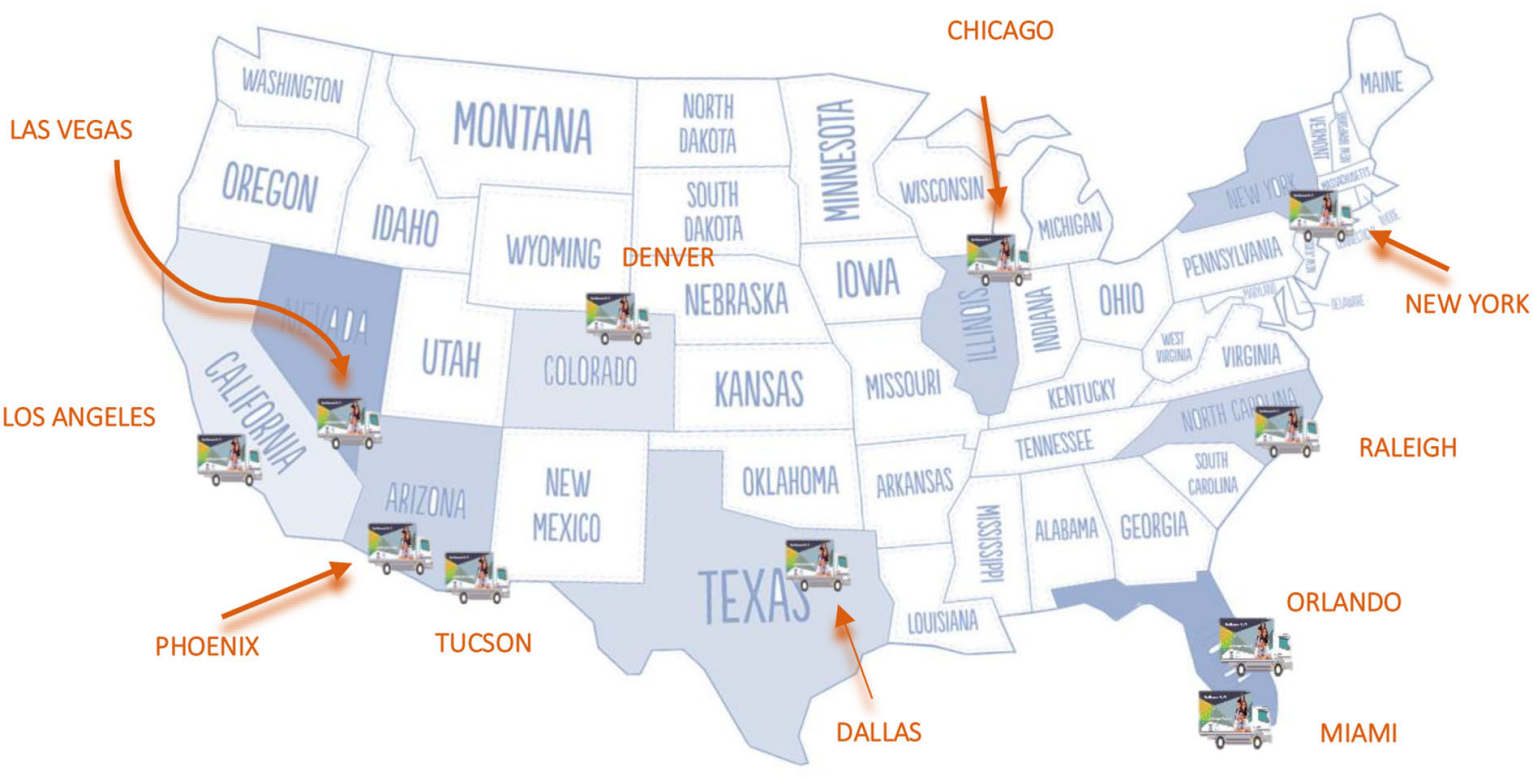
Geographical location of Mobile Health Units in the United States. Source: Mexican section of the Mexico-United States Border Health Commission, 2020.

To strengthen the VDS, the Mobile Health Units's (MHU) care model was created to provide preventive health services to remote communities with difficult access to health services. Eleven MHU currently operate in cities with a large concentration of Hispanic populations: Chicago, Dallas, Denver, Las Vegas, Los Angeles, Miami, New York, Orlando, Phoenix, Raleigh, and Tucson (Map 2).

### Background

The VDS strategy was initiated in response to the success of the Binational Health Week and its acceptance by the community. During this event, preventive and health promotion services are offered annually to Hispanics who typically experience barriers to accessing such services in the United States. Such improved access and acceptance led community leaders and local organizations to request long term continuity of these services. In 2003, the VDS strategy was implemented as a pilot project at the Mexican Consulates in San Diego and Los Angeles, California, with the support of the United States-Mexico Border Health Commission and the United States-Mexico Health initiative (known as the Health Initiative of the Americas), from the University of California and The California Endowment. In 2004, the VDS strategy was formally launched and extended to other consulates. With the aim of strengthening and expanding the coverage of preventive health services for the Mexican community in the United States to remote communities unable to access health services, in 2016, the first phase of the MHU care model was implemented in Dallas, Los Angeles, New York, Phoenix and Chicago. Its services were subsequently extended to Denver, Las Vegas, Raleigh, Orlando, Miami, and Tucson (7).

### VDS Strategy Characteristics

The VDS strategy is an example of government and institutional collaboration with strategic allies in Mexico and the United States. The resources, both monetary and in kind, required to provide basic preventive services, are contributed by the following actors:

- Mexican goverment
  - Seed money
  - Institutional collaboration
- Consular network
  - Space on their premises
  - Technology and support office
  - Alliances
  - Training
- Lead agencies
  - Manage and operate the VDS
  - Select and hire staff
  - Generate intervention projects
  - Organize fairs and events
- Network of associated agencies
  - Provide specialied personnel
  - Perform screening tests and administration fof vaccines
  - Carry out workshops and deliver educational materials and workshops
  - Participate in health fairs and training

The VDS strategy has an advisory board comprised of nine members, including leaders in the health and migrant assistance sectors, who represent different sectors: government, academia, the non-government sector, and international organizations, from Mexico and the United States. The purpose of the Advisory Board is to provide advice on management, innovation, and binational managerial strengthening processes with the various sectors to improve strategy (8).

In general, VDS and MHU focus on conditions with the highest incidence rates among the Mexican population living in the United States, grouping services into five categories:

1) ***Counseling*** on prevention and health promotion issues through advice on priority health issues, such as nutrition, obesity, diabetes, women's health, child health, mental health, addictions, HIV/AIDS, and access to services, among others.
2) ***Timely detection*** of various ailments, using measurements such as body mass index, cholesterol and glucose tests, and HIV/AIDS and COVID-19 tests.
3) ***Referrals*** to health services available in their locality (community clinics), related to cultural and language particularities, to receive medical care if necessary and/or establish a medical home.
4) ***Administration of flu and COVID-19 vaccines*** and for other illnesses.
5) ***Advice*** on health insurance alternatives in the United States.

### Educational and technological tools

Activities undertaken by VDS and MHU are based on training health promoters in priority health issues to strengthen their skills and provide comprehensive preventive health services for users (9). During 2020 and the first semester of 2021, various training sessions and webinars were held whose main topics were COVID-19 and mental health.

In addition, these activities were supported using culturally adapted educational materials in Spanish focusing on health promotion and disease prevention. Between 2020 and the first semester of 2021, technical content was developed on various priority health topics (such as healthy lifestyle; chronic-degenerative diseases; health promotion; sexual and reproductive health; child health; mental health and COVID-19), which subsequently allowed the design of 301 educational materials. The material is disseminated in community events and through social media networks: Facebook, Twitter, Instagram, YouTube, and the VDS strategy website.

Likewise, in 2018, the Mexican Section of USMBHC launched the mental health initiative. In collaboration with the Pan American Health Organization (PAHO), it implemented training in the Mental Health Gap Action Programme (mhGAP) Intervention Guide designed to help health personnel and community health workers (promotores de salud) reduce gaps in mental health. The training covers issues such as risk factors and warning signs of mental illness and information on depression, anxiety, trauma, and psychosis either to help a person cope with the mental health problem and/or to refer them in a timely manner to professional help [(10), p. 14].

SICRESAL-MX, the data collection system previously described in the methods section, is the official mechanism for registering users of VDS and MHU services and for generating real-time quantitative data describing the sociodemographic and health conditions of users and the VDS and MHU services received by users. The database is a rich source of information with about 283 variables. Its importance lies in the fact that the capture of such data through the registration process enables its analysis through descriptive statistics for decision-making and the strengthening of preventive health services.

### Impact

The VDS strategy has met the needs of their users by providing comprehensive health services through culturally adapted preventive health promotion actions, by contributing to disease control, and preventing the use of emergency service through timely detection.

The MHU care model has taken these services to remote communities that are unable to access health services, thus, reaching a sector of a vulnerable community and broadening the scope of services.

According to data from SICRESAL-MX, between 2019 and 2021, the VDS and MHU served approximately 6.4 million people and provided 20.7 million services, of which 88.2% corresponded to counseling or advice, 8.1% to screening, 3.1% to vaccination services, 0.2% to information on other issues, and 0.1% to referrals. Tables 1, 2 shows the disaggregation of services and people served by type of strategy, year, and service.

**Table 1:** Population served and services provided at VDS, January 2019—December 2021.

**Year**	**Population served**	**Total services provided**	Advice/counseling*	**Information on other issues**	**Screening**	**Vaccines**	**Referrals**
2019	1,394,159	3,918,773	3,208,957	22,203	593,655	86,055	7,903
2020	3,060,946	8,582,100	8,166,127	5,747	402,908	51,234	6,084
2021	1,319,481	5,014,824	4,132,432	5,994	490,021	382,830	7,937
Total	5,774,586	17,515,697	15,507,516	33,944	1,486,584	520,119	21,924

Source: Compiled by the author based on data from the SICRESAL-MX. *In 2020, in the face of the COVID-19 pandemic and social distancing as a preventive measure, the VDS and MHU developed a communication system through social networks to continuously provide guidance and education services. At the same time, SICRESAL-MX adapted to this need and integrated the dissemination of information through social networks, as part of VDS andMHU counseling services notably increasing the numbers reported. As of April 2021, the registration for in-person counseling services was separated from the work of disseminating information on social networks, to have clarity of the work carried out by VDS and MHU by type of service.

**Table 2:** Population served and services provided in MHU, January 2019—December 2021.

**Year**	**Population served**	**Total services provided**	Advice/counseling^*^	**Information on other issues**	**Screening**	**Vaccines**	**Referrals**
2019	28,631	164,772	96,408	7,416	54,890	4,188	1,870
2020	295,160	1,161,157	1,017,979	922	35,778	6,059	429
2021	297,918	1,858,412	1,640,099	2,300	99,143	101,444	396
Total	621,709	3,184,341	2,754,486	10,638	189,811	111,691	2,695

Source: Compiled by the author based on data from SICRESAL-MX.

* In 2020, in the face of the COVID-19 pandemic and social distancing as a preventive measure, the VDS and MHU developed a communication system through social networks to continuously provide guidance and education services. At the same time, SICRESAL-MX adapted to this need and integrated the dissemination of information through social networks, as part of VDS and MHU counseling services notably increasing the numbers reported. As of April 2021, the registration for in-person counseling services was separated from the work of disseminating information on social networks, to have clarity of the work carried out by VDS and MHU by type of service.

This level of care has achieved a favorable impact on the health of Hispanic VDS service community in the United States by offering preventive services that enable identifying ailments and raising health issue awareness. In addition, it contributes to disease control and prevents the use of emergency services through timely detection, service availability advice, and aiding in the establishment of a medical home. Likewise, because of the COVID-19 pandemic, services were expanded to adapt to the new context.

Of those receiving screening services for specific conditions at the VDS and MHU, the most important data are presented below (Table 3):

- Glucose screening showed that two out of every 10 people screened at VDS and three out of 10 at MHU displayed high levels.
- An alarming piece of data is that around three-quarters of people screened in VDS and MHU have overweight and obesity problems, especially men.
- In general, three out of 10 users attended VDS and MHU had high cholesterol levels. Once again, a higher percentage of men experienced this condition compared with women, especially among those who attended VDS.
- While six of every 10 persons had high blood pressure, women experienced a higher prevalence than men.

**Table 3:** Percentage distribution of results of anthropometric measurement and glucose, cholesterol, and blood pressure tests, of the population served at VDS and MHU, by sex and type of strategy, January 2019—December 2021^a^.

**(a) Ventanillas de Salud**				**(b) Mobile health units**			
**Glucose level**	**Men**	**Women**	**Total**	**Glucose level**	**Men**	**Women**	**Total**
High	22%	20%	21%	High	29%	25%	26%
Low	1%	3%	2%	Low	1%	1%	1%
*n* = 26,118 cases				*n =* 10,210 cases			
**Anthropometric measurements**	**Men**	**Women**	**Total**	**Anthropometric measurements**	**Men**	**Women**	**Total**
Overweight/Obesity	76%	69%	72%	Overweight/Obesity	77%	75%	76%
Low weight	1%	1%	1%	Low weight	0%	1%	0%
*n =* 25,884 cases				*n =* 10,416 cases			
**Cholesterol level**	**Men**	**Women**	**Total**	**Cholesterol level**	**Men**	**Women**	**Total**
High	37%	29%	33%	High	24%	30%	27%
Low	5%	8%	7%	Low	2%	1%	1%
*n =* 5,263 cases				*n =* 2,421 cases			
**Blood pressure level**	**Men**	**Women**	**Total**	**Blood pressure level**	**Men**	**Women**	**Total**
High	53%	66%	60%	High	52%	63%	59%
Low	45%	29%	37%	Low	47%	35%	40%
*n =* 34,433 cases				*n =* 12,792 cases			

Source: Compiled by the author based on data from the SICRESAL-MX.

a To categorize screening results, the VDS strategy uses the following sources.

Academia Estadounidense de Médicos Familiares, en https://es.familydoctor.org/perfil-de-lipidos-en-el-analisis-de-sangre/.

Biblioteca Nacional de Medicina, Institutos Nacionales de Salud de los Estados Unidos, en https://medlineplus.gov/spanish/ency/patientinstructions/000386.htm.

Biblioteca Nacional de Medicina, Institutos Nacionales de Salud de los Estados Unidos, en https://medlineplus.gov/spanish/cholesterollevelswhatyouneedtoknow.html.

During the period of analysis (2019–2021), data from the SICRESAL-MX related to family history of illness of 41,000 people receiving either VDS and/or MHU services showed that 55% have a family history of diabetes and 20% have a family history of obesity. In addition, 452 mental health screenings were performed during this period indicating the prevalence of the following: depression (40%), anxiety (16%), psychosis (14%), violence (8%), sexual problems (8%), and other more rare conditions (14%).

During the period of analysis, just over 154,000 people were served at VDS and MHU and provided information on their medical coverage (Table 4). These data show that in general this is a highly vulnerable population since 94% lack any type of health coverage, and more so among VDS (96%) than MHU users (85%). Moreover, when separated by age group, people aged 65 or over have the highest percentage of medical coverage, especially among MHU users.

**Table 4:** Percentage distribution (in lines and columns) of population served at VDS and MHU by age group and medical insurance status, January 2019—December 2021.

**Percentage distribution in lines (by age group)**	**Age groups**	**MHU** **(*n* = 27,794)**	**VDS** **(*n* = 126,470)**
		**Uninsured**	**Uninsured**
	Under 5	85%	98%
	5 to 9	97%	94%
	10 to 17	89%	95%
	18 to 30	88%	97%
	31 to 40	87%	98%
	41 to 50	88%	97%
	51 to 65	82%	94%
	Over 65	60%	87%

Source: Compiled by the author based on data from SICRESAL-MX.

## Discussion

The services provided by VDS and MHU in the United States are crucial for several reasons:

First, there is a large and growing size of Hispanic population living in the United States. According to data from the most recent Census conducted in 2020, the total Hispanic population living in the United States amounted to 62.1 million, of whom approximately 62% were of Mexican origin (38.5 million people).

Second, it addresses the predominant characteristics that act as barriers to medical care among this population such as their economic status, limited command of the English language, and limited access to health services that result in them suffering from poorer health.

Third, the VDS and MHU address conditions that stem from a history of chronic-degenerative illnesses and diseases among the Mexican population, such as obesity, diabetes, and hypertension, documented both in Mexico, through the National Survey of Health and Nutrition (11), and the United States through the National Health Interview Survey (12).

Fourth, in recent years mental health has become a major concern in offering preventive and holistic care and giving it the same level of care as physical health problems receive. VDS and MHU began an initiative to approach healthcare among Mexicans in the United States to detect mental health signs and symptoms, and to refer them to access local or online services.

Fifth in the face of the COVID-19 pandemic, VDS and MHU adapted its services and system and type of care offered to continue providing in person preventive services, such as administering vaccines, also, providing remote services, showing their ability to adapt and respond to emerging needs.

Given these characteristics and based on the results of the quantitative analysis, both VDS and MHU are considered successful models in managing to detect the specific needs of their users and adapting to the context where they are implemented. They have also been strengthened over the years, thanks to their network of strategic allies in each consular district, which in turn manifests their perseverance, coordination, and institutional commitment.

## Conclusion

VDS and MHU have proven to be an important model of collaboration. Through alliances with institutions from different sectors, VDS and MHU have managed to direct outreach to a vulnerable sector of the Hispanic population to reduce the gap in access to health services. It is a unique care model, serving co-nationals in another country, contributing not only to the benefit of the health and wellness of Mexicans in the United States, but also to the health and wellness of other neighboring Hispanics.

The ability of the VDS/MHU to effectively organize and manage health outreach services and to connect clients to these services, particularly in the area of COVID-19 and mental health, is a testament to the strength of its diverse network of collaborators in Mexico and the United States.

In conclusion, we would like to suggest the following actions to strengthen the VDS and MHU services and ensure the continuity of the strategy. First, VDS and MHU should identify and formalize collaborations with strategic allies to promote the supply and distribution of the COVID-19 vaccine and others vaccines to the neediest and most vulnerable migrant communities. Second, it is also important to strengthen the mental health module. The relevancy of this module was highlighted by the COVID-19 pandemic and the detrimental impact on mental health related to social distancing and sheltering in place. Third, the VDS and MHU requires greater dissemination of the work carried out to strengthen their actions and give continuity to the strategy.

The VDS strategy has been instrumental in providing much needed health-related preventive services to hard-to-reach populations and communities which they would otherwise have been unable to obtain.

## Data availability statement

The datasets generated for this study are available on reasonable request to the corresponding author.

## Author contributions

IL and SS first draft and data analysis. MR, AL, AR, MC, LG, and CR revision and final draft. All authors contributed to the article and approved the submitted version.

## Funding

Initial funding for the project provided by the Mexican Government.

## Conflict of interest

The authors declare that the research was conducted in the absence of any commercial or financial relationships that could be construed as a potential conflict of interest. The reviewer KD declared a past co-authorship with the authors MG and CR to the handling editor.

## Publisher's note

All claims expressed in this article are solely those of the authors and do not necessarily represent those of their affiliated organizations, or those of the publisher, the editors and the reviewers. Any product that may be evaluated in this article, or claim that may be made by its manufacturer, is not guaranteed or endorsed by the publisher.
